# Supplementary material for: Inhibitory Effects of Chlorogenic Acid Containing Green Coffee Bean Extract on Lipopolysaccharide-Induced Inflammatory Responses and Progression of Colon Cancer Cell Line
Source: Foods. 2023 Jul 9;12(14):2648. doi: 10.3390/foods12142648 (PMC10378980; doi:10.3390/foods12142648)

Supplementary material

Western blot image

Fig. 3A

TLR4 (lane 1: NoPLS, lane 2: LPS, lane 3: LPS+EIF25, lane 4: LPS+EIF50, lane 5: LPS+EIF75, lane 6: LPS+CGA5, lane 7: LPS+CGA10, lane 8: LPS+CGA20)

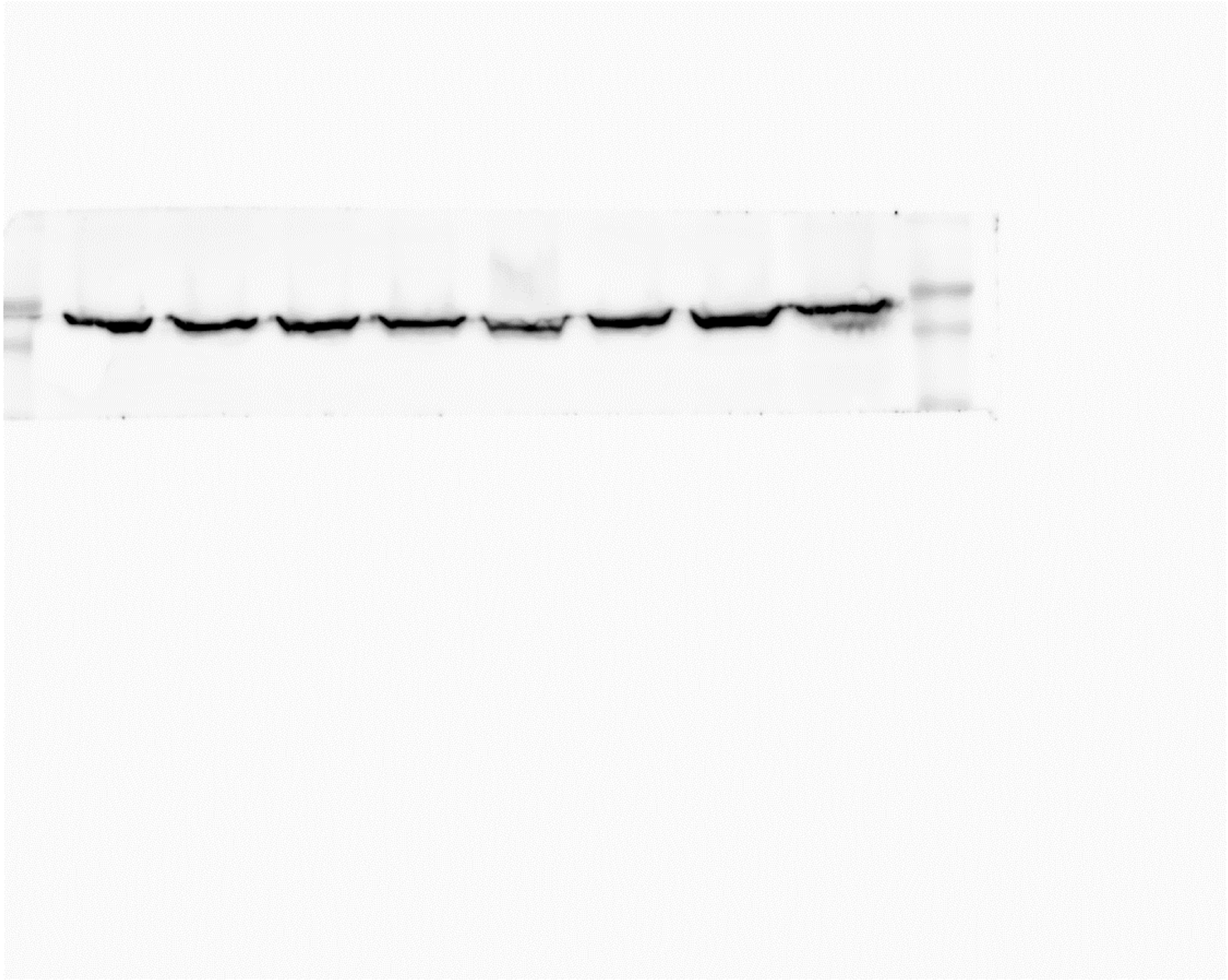

Fig. 3A

B-actin (lane 1: NoPLS, lane 2: LPS, lane 3: LPS+EIF25, lane 4: LPS+EIF50, lane 5: LPS+EIF75, lane 6: LPS+CGA5, lane 7: LPS+CGA10, lane 8: LPS+CGA20)

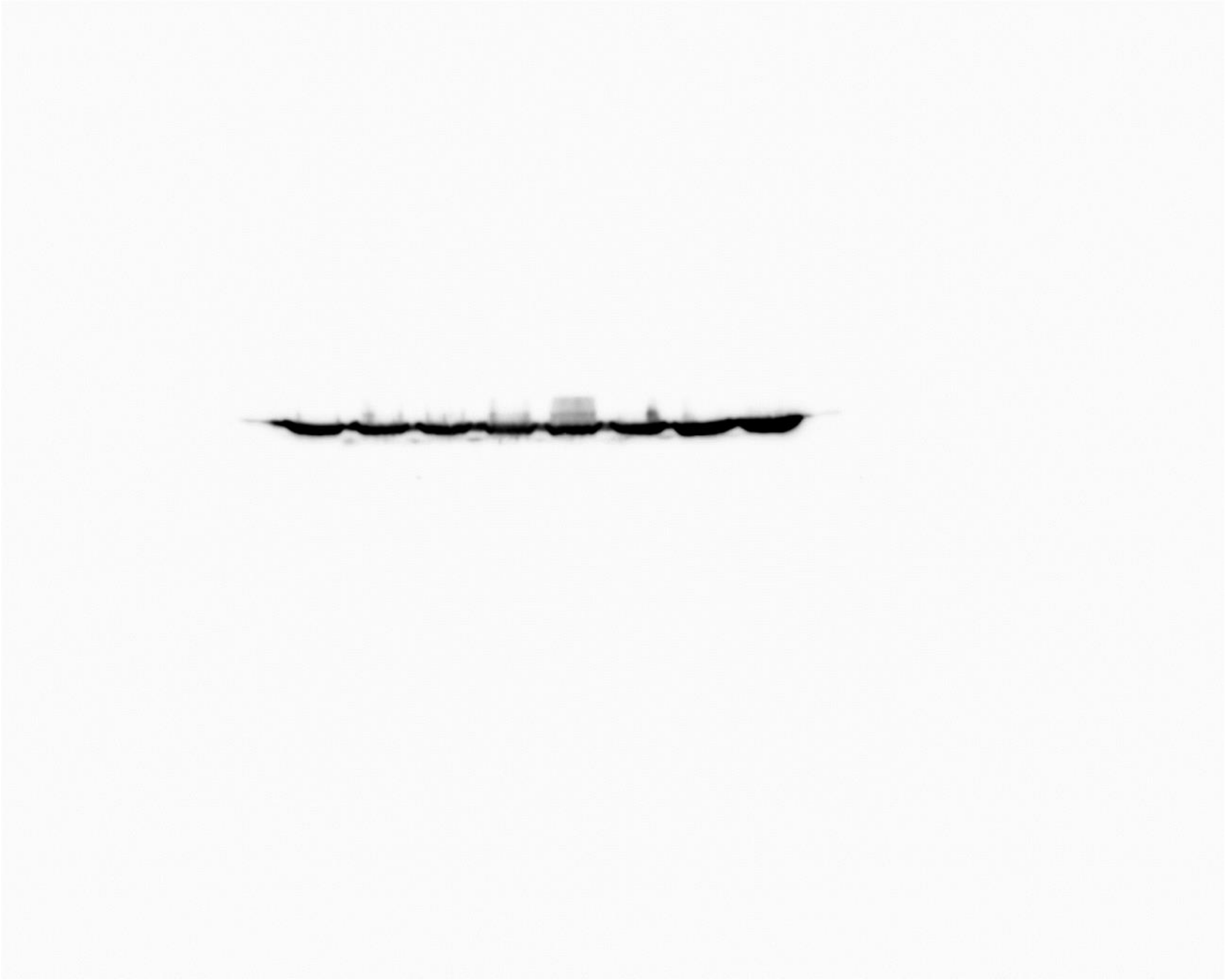

Fig. 3C

<u>TLR4</u> (lane 1: NoPLS, lane 2: LPS, lane 3: LPS+ESF25, lane 4: LPS+ESF50, lane 5: LPS+ESF75, lane 6: LPS+CF20, lane 7: LPS+CF40, lane 8: LPS+CF60)

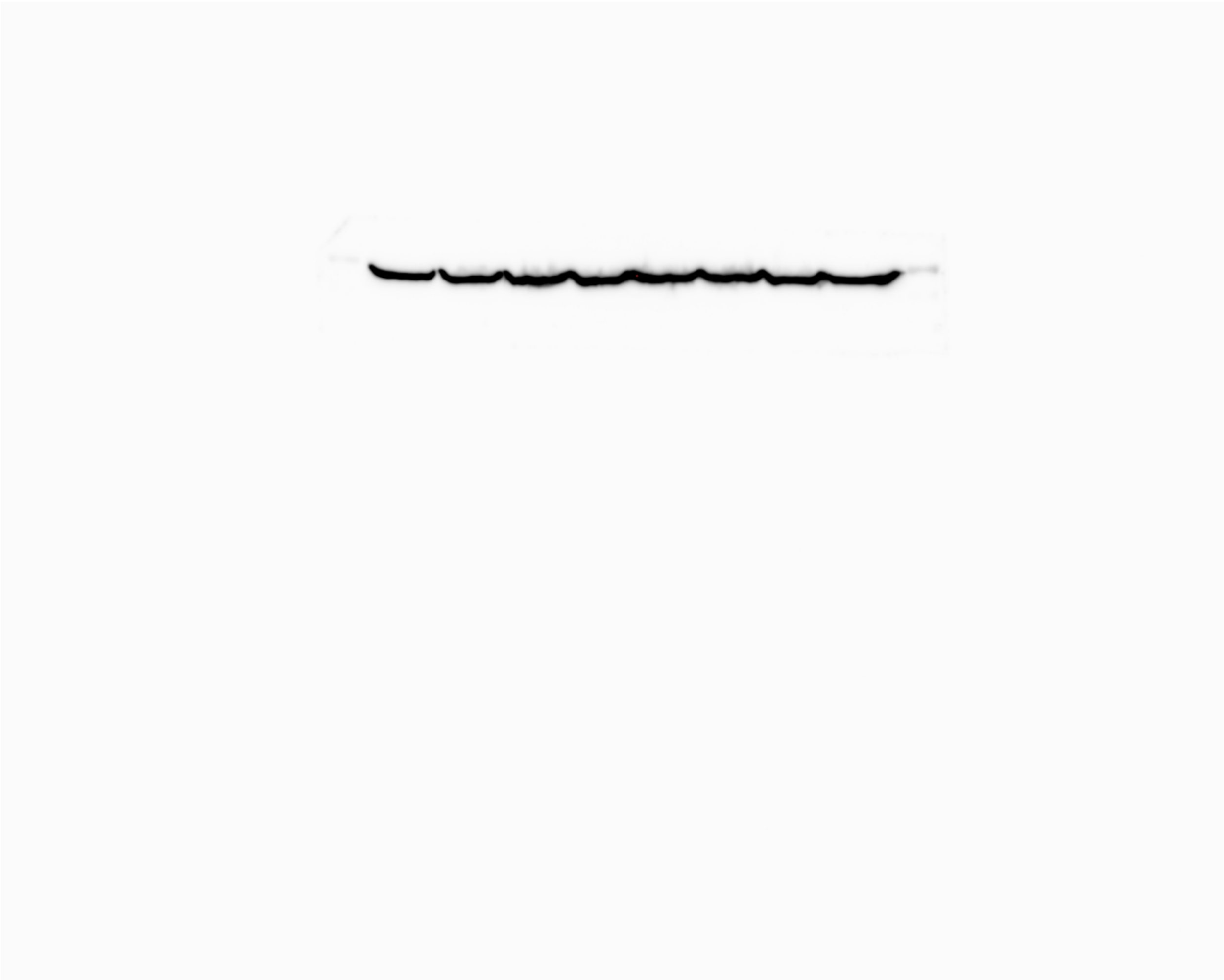

Fig. 3C

<u>B-actin</u> (lane 1: NoPLS, lane 2: LPS, lane 3: LPS+ESF25, lane 4: LPS+ESF50, lane 5: LPS+ESF75, lane 6: LPS+CF20, lane 7: LPS+CF40, lane 8: LPS+CF60)

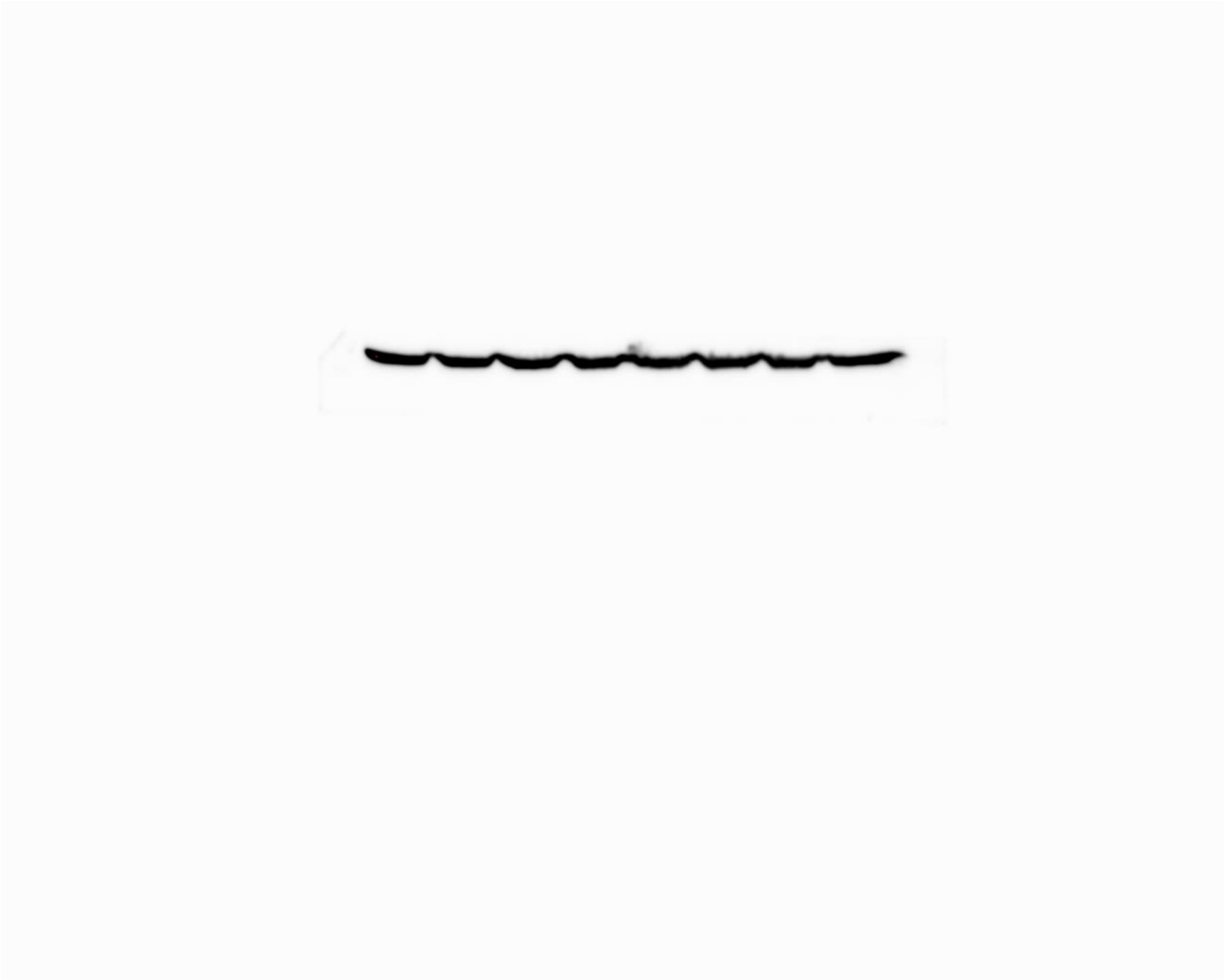

Fig. 4A

<u>**COX-2**</u> (lane 1: NoPLS, lane 2: LPS, lane 3: LPS+EIF25, lane 4: LPS+EIF50, lane 5: LPS+EIF75, lane 6: LSP+CGA5, lane 7: LPS+CGA10, lane 8: LPS+CGA20)

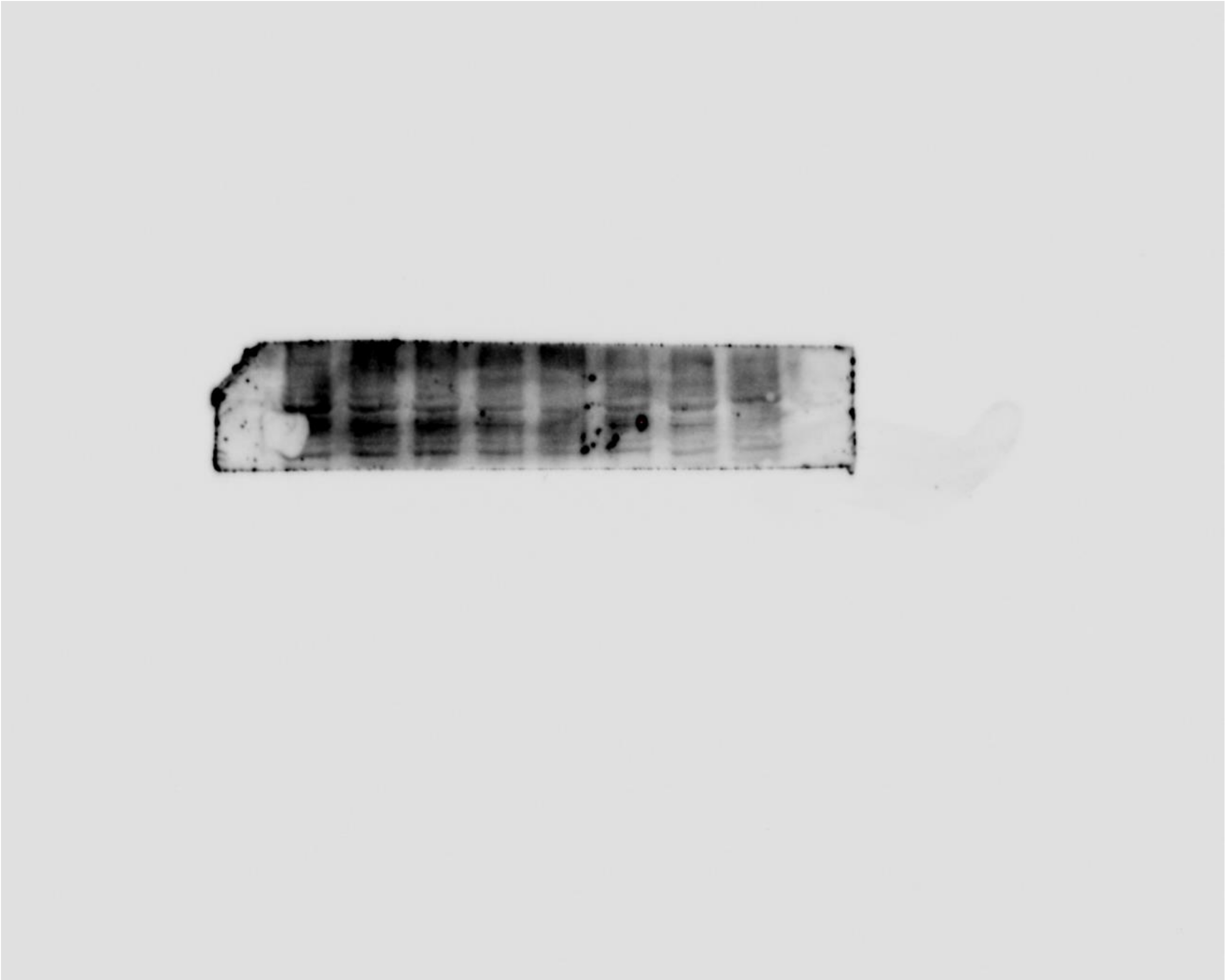

Fig. 4A

B-actin (lane 1: NoPLS, lane 2: LPS, lane 3: LPS+EIF25, lane 4: LPS+EIF50, lane 5: LPS+EIF75, lane 6: LSP+CGA5, lane 7: LPS+CGA10, lane 8: LPS+CGA20)

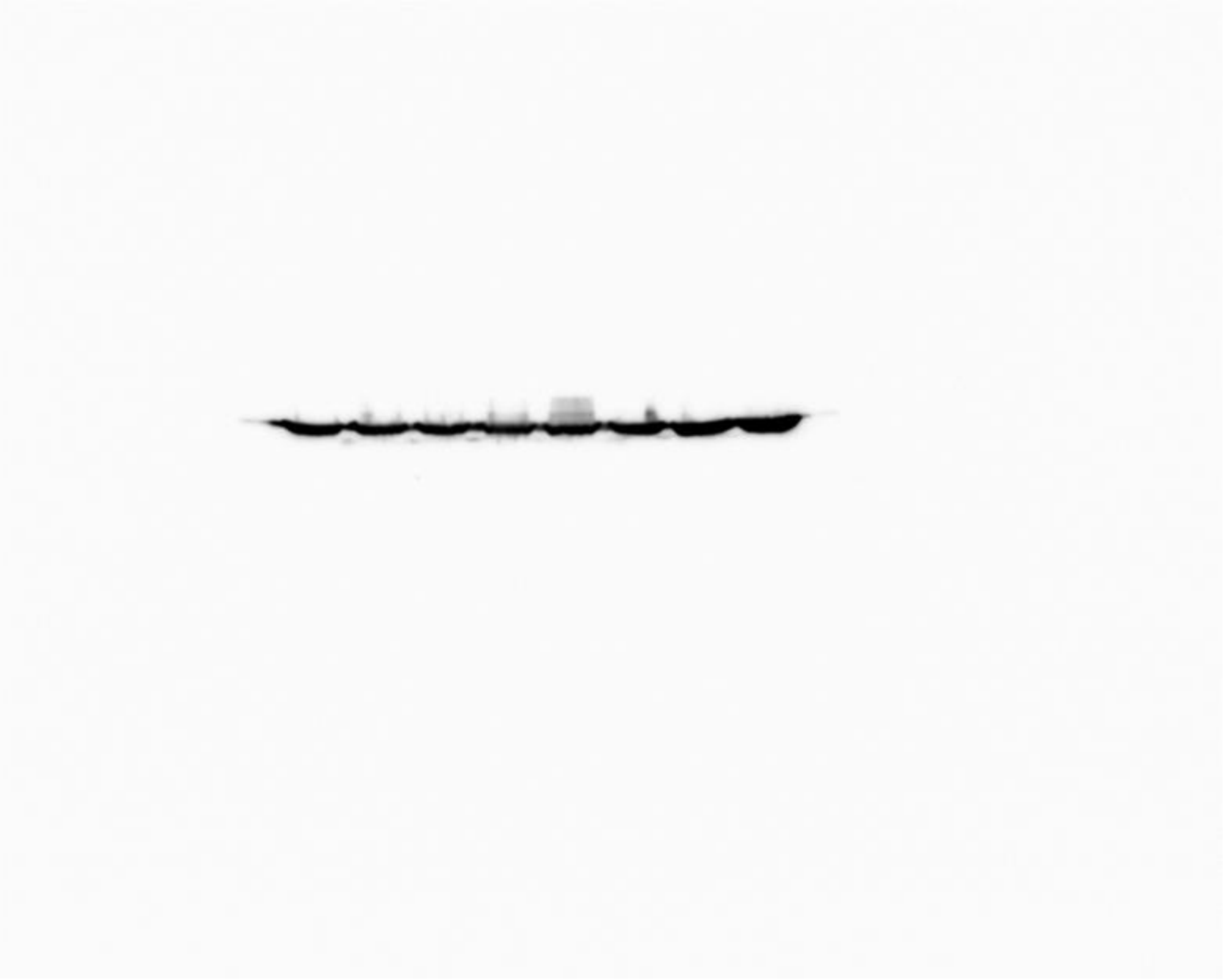

Fig. 4A

<u>TNF-alpha</u> (lane 1: NoPLS, lane 2: LPS, lane 3: LPS+EIF25, lane 4: LPS+EIF50, lane 5: LPS+EIF75, lane 6: LPS+CGA5, lane 7: LPS+CGA10, lane 8: LPS+CGA20)

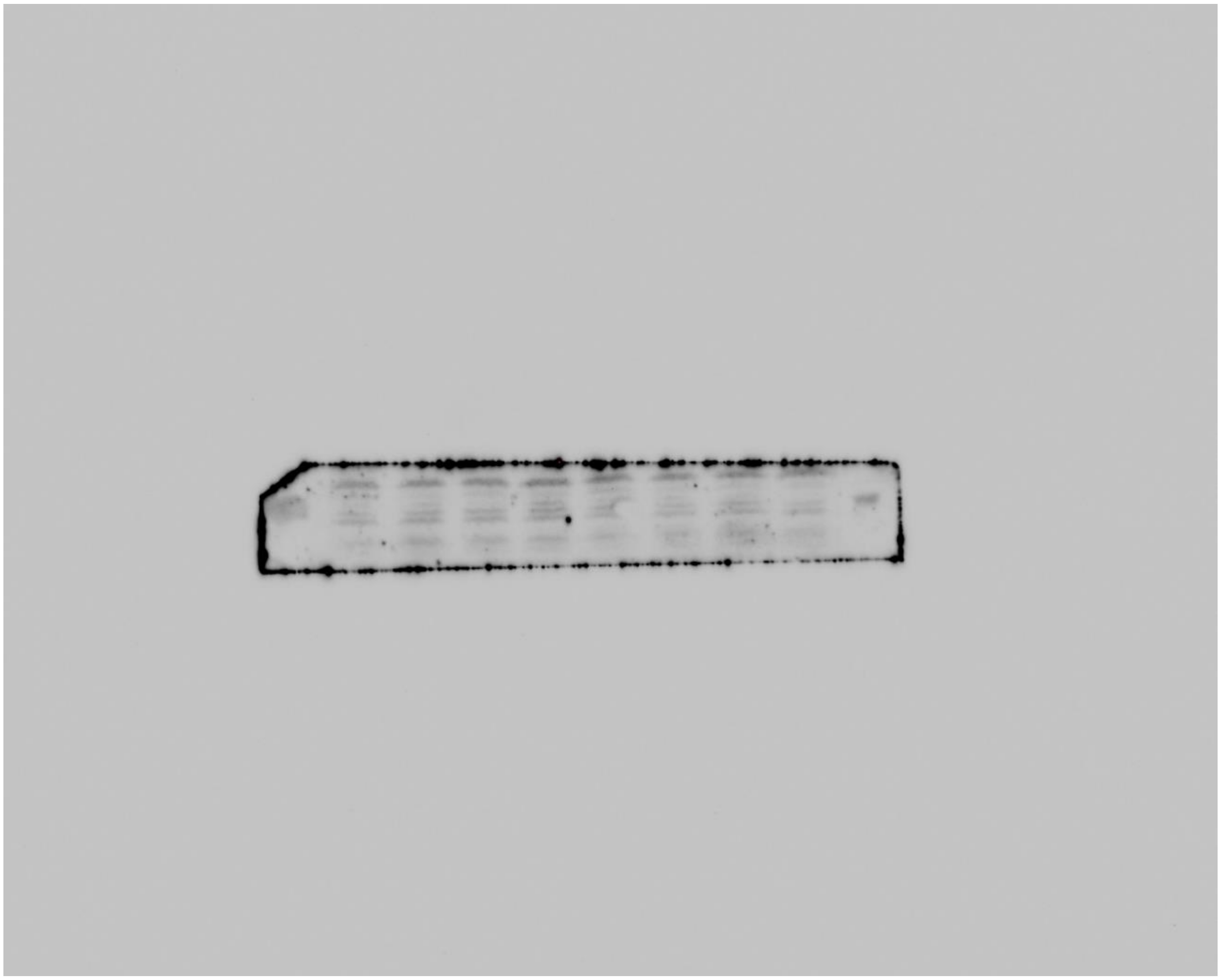

Fig. 4A

B-actin (lane 1: NoPLS, lane 2: LPS, lane 3: LPS+EIF25, lane 4: LPS+EIF50, lane 5: LPS+EIF75, lane 6: LSP+CGA5, lane 7: LPS+CGA10, lane 8: LPS+CGA20)

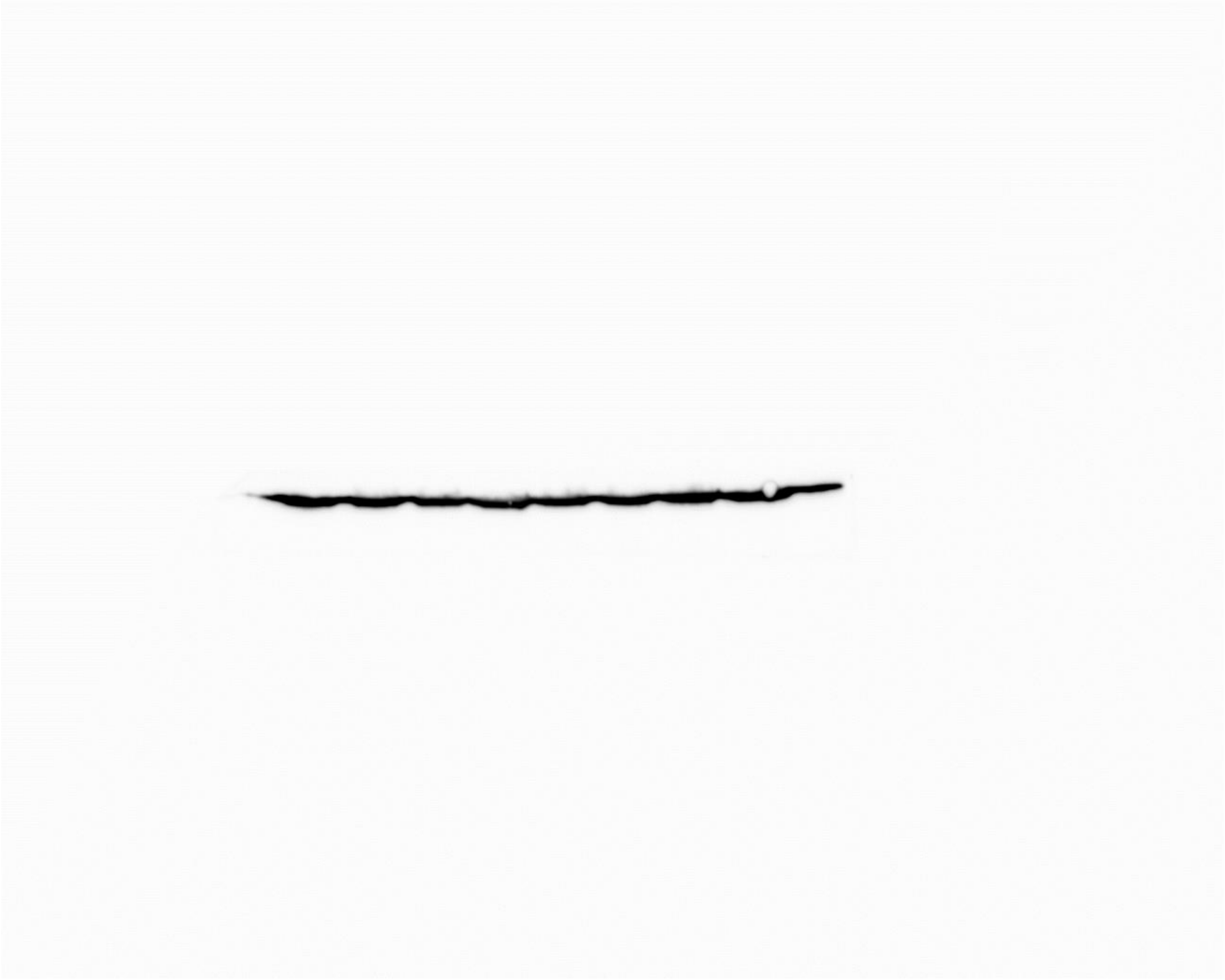

Fig. 4A

<u>IL-1β</u> (lane 1: NoPLS, lane 2: LPS, lane 3: LPS+EIF25, lane 4: LPS+EIF50, lane 5: LPS+EIF75, lane 6: LSP+CGA5, lane 7: LPS+CGA10, lane 8: LPS+CGA20)

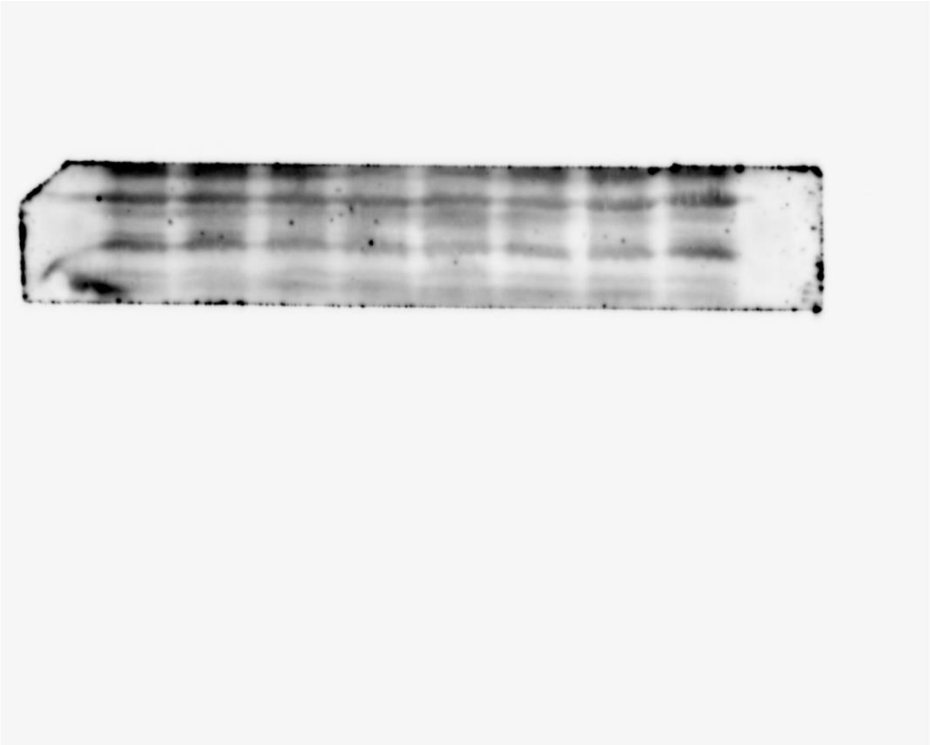

Fig. 4A

<u>B-actin</u> (lane 1: NoPLS, lane 2: LPS, lane 3: LPS+EIF25, lane 4: LPS+EIF50, lane 5: LPS+EIF75, lane 6: LSP+CGA5, lane 7: LPS+CGA10, lane 8: LPS+CGA20)

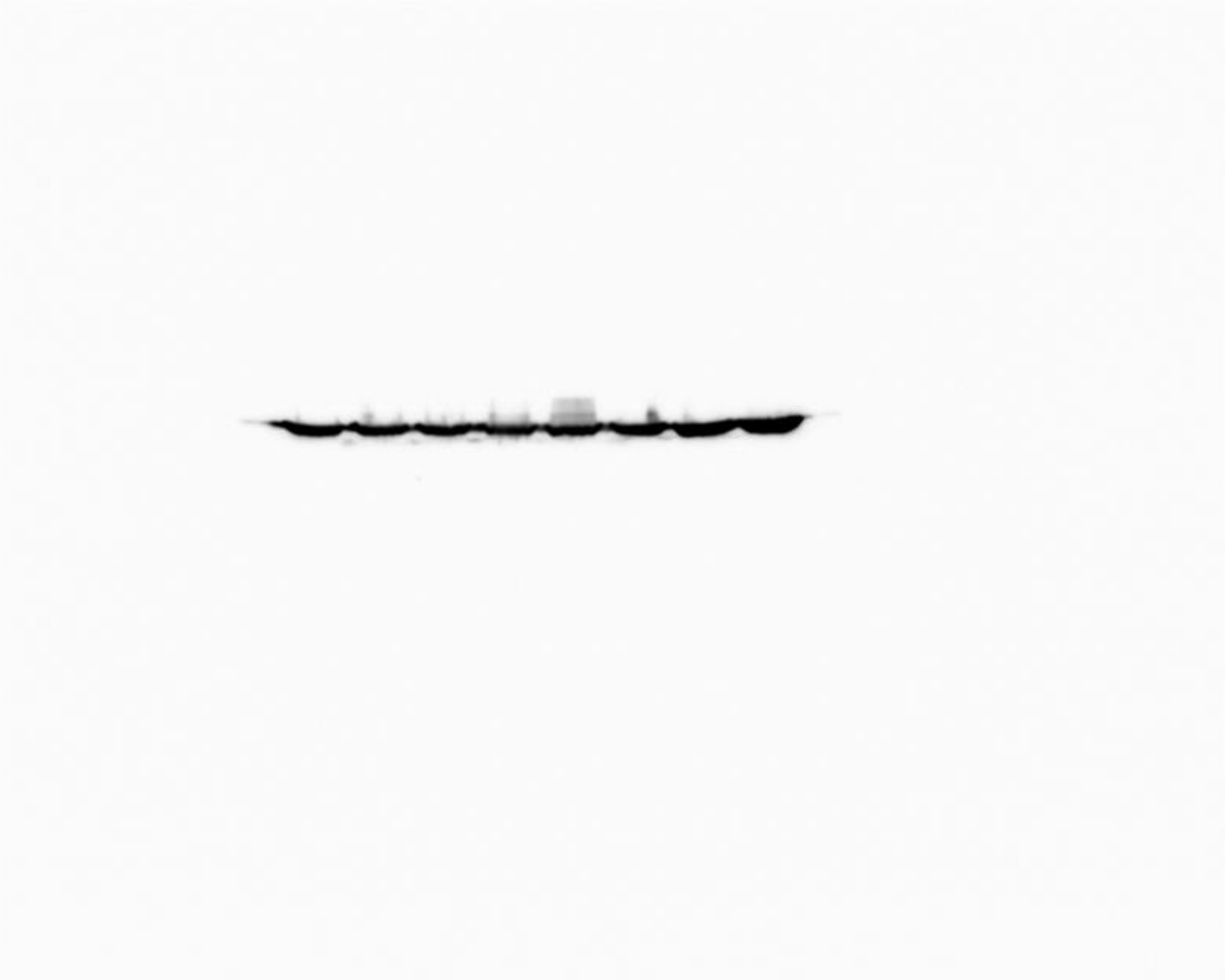

Fig. 5A

<u>VEGFC</u> (lane 1: NoPLS, lane 2: LPS, lane 3: LPS+EIF25, lane 4: LPS+EIF50, lane 5: LPS+EIF75, lane 6: LSP+CGA5, lane 7: LPS+CGA10, lane 8: LPS+CGA20)

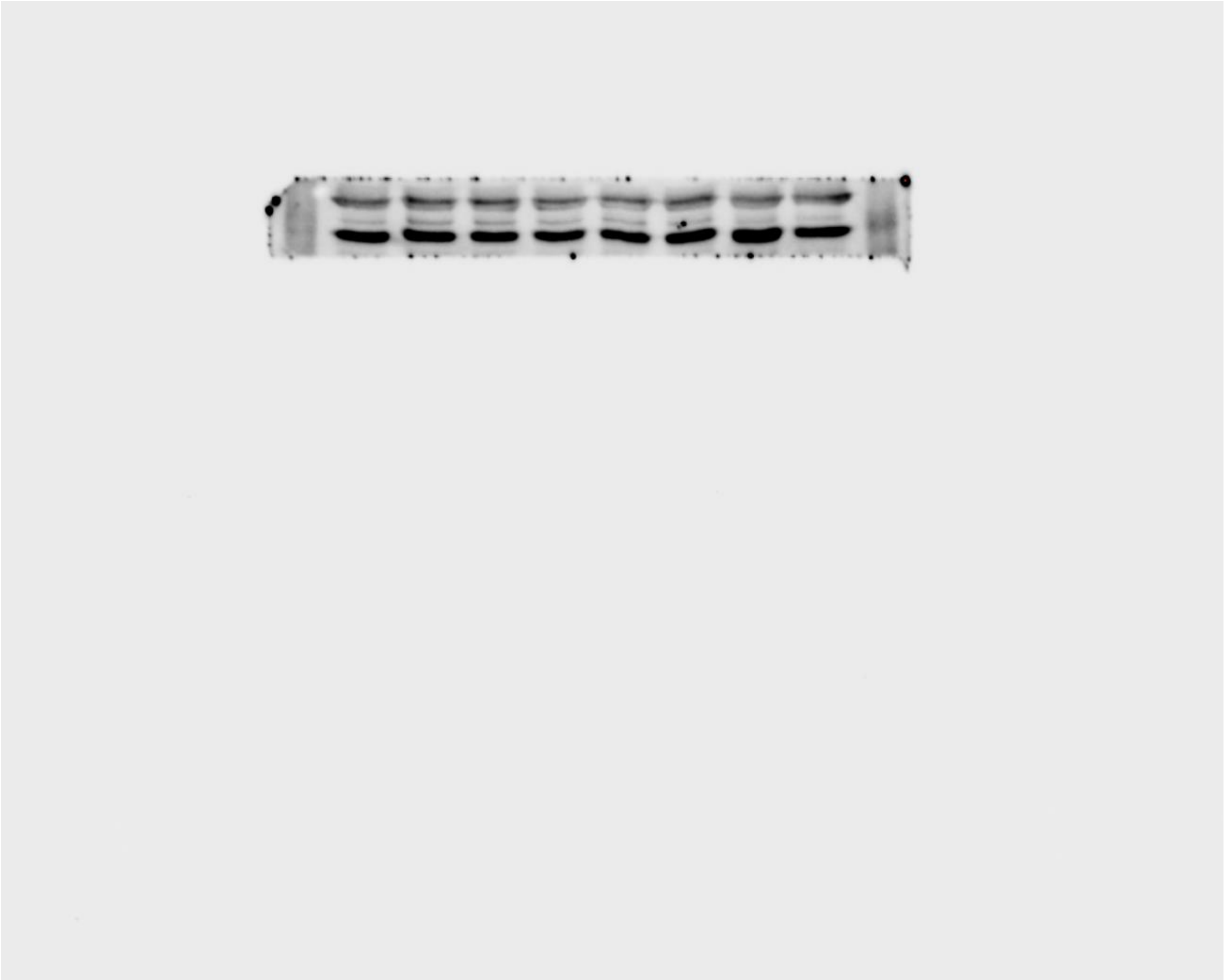

Fig. 5A

B-actin (lane 1: NoPLS, lane 2: LPS, lane 3: LPS+EIF25, lane 4: LPS+EIF50, lane 5: LPS+EIF75, lane 6: LSP+CGA5, lane 7: LPS+CGA10, lane 8: LPS+CGA20)

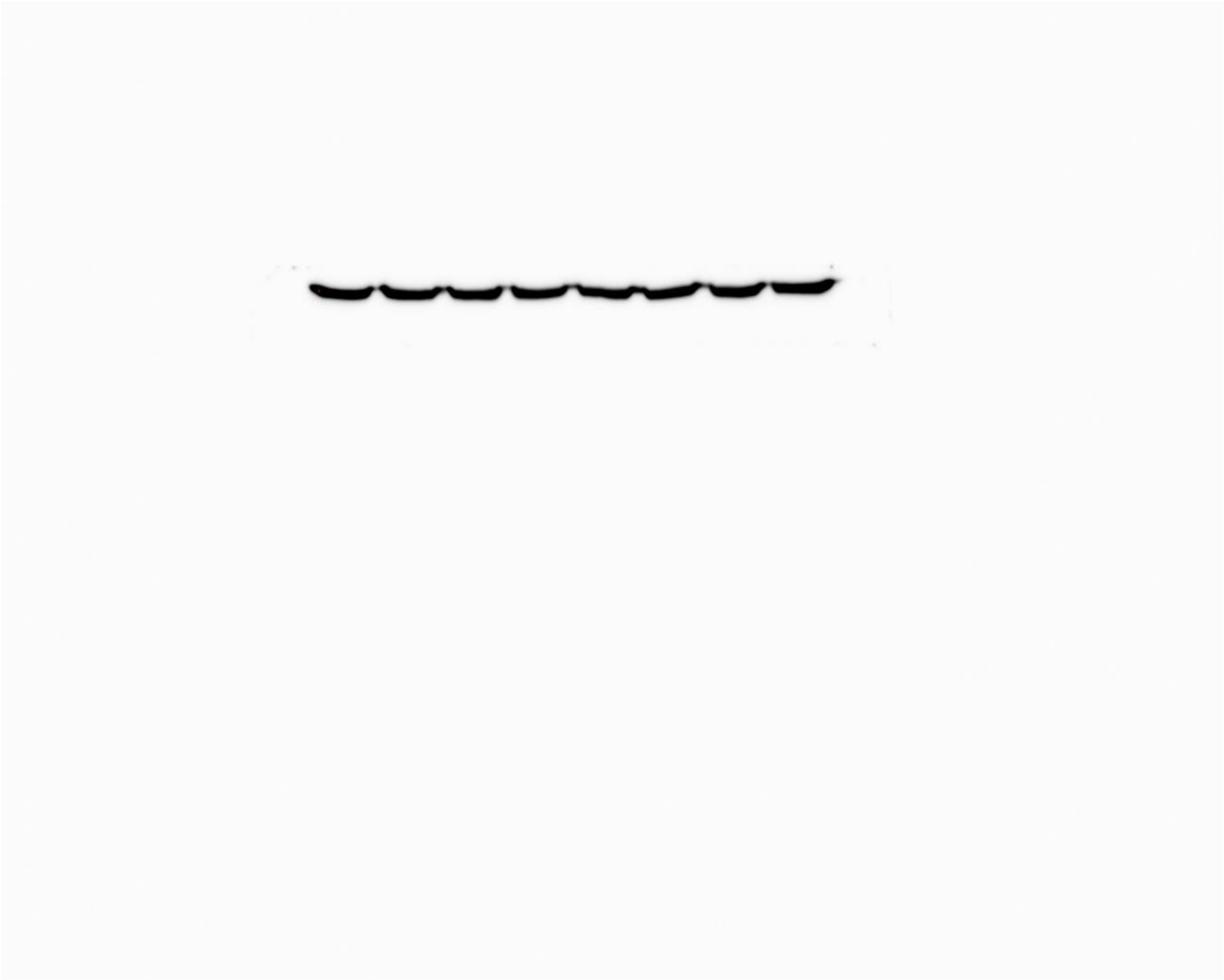

Supplement: Supplementary file 1 [file foods-12-02648-s001.zip › foods-2490361-supplementary.pdf]
